# Phylogeny of *Camphora* and *Cinnamomum* (Lauraceae) Based on Plastome and Nuclear Ribosomal DNA Data

**DOI:** 10.3390/ijms26031370

**Published:** 2025-02-06

**Authors:** Jian Xu, Haorong Zhang, Fan Yang, Wen Zhu, Qishao Li, Zhengying Cao, Yu Song, Peiyao Xin

**Affiliations:** 1 Engineering Technology Research Center of National Forestry and Grassland Administration on Southwest Landscape Architecture, Southwest Forestry University, Kunming 650224, China; xujian960128@163.com (J.X.); zhr23122x@163.com (H.Z.); yf@swfu.edu.cn (F.Y.); zhuwen@swfu.edu.cn (W.Z.); 15308895069@163.com (Q.L.); czy1808@163.com (Z.C.); 2 Key Laboratory of Ecology of Rare and Endangered Species and Environmental Protection (Ministry of Education) & Guangxi Key Laboratory of Landscape Resources Conservation and Sustainable Utilization in Lijiang River Basin, Guangxi Normal University, Guilin 541001, China

**Keywords:** *Camphora*, *Cinnamomum*, plastome, sequence characteristic, phylogenetic analysis

## Abstract

*Camphora* Fabr. is a genus in the family Lauraceae, comprising over 20 tropical and subtropical tree species. Since the genera *Camphora* and *Cinnamomum* Schaeff. were described, there has been a long-lasting controversy regarding the phylogenetic relationships among taxa in both genera. In particular, phylogenetic inferences derived from plastid data remain debated, with varying hypotheses proposed and occasional disputes concerning the monophyly of *Camphora* taxa. To further investigate the relationships, We analyzed plastomes and nuclear ribosomal cistron sequences (nrDNA) of 22 *Camphora* taxa, 15 *Cinnamomum* taxa, and 13 representative taxa of related genera. The *Camphora* plastomes range from 152,745 to 154,190 bp, with a GC content of 39.1% to 39.2%. A total of 128 genes were identified in the *Camphora* plastomes, including 84 protein-coding genes, 8 rRNA genes, and 36 tRNA genes. A total of 1130 SSR loci were detected from plastomes of *Camphora*, and A/T base repeats looked like the most common. Comparative analyses revealed that the plastomes of *Camphora* exhibit high similarity in overall structure. The loci *ycf1*, *ycf2*, *trnK (UUU)*, *psbJ-psbL*, and *ccsA-ndhD* were identified as candidate DNA barcodes for these taxa. Plastome phylogenetic analysis revealed that *Camphora* is not monophyletic, whereas the nrDNA dataset supported the monophyly of *Camphora*. We propose that intergeneric hybridization may underlie the observed discordance between plastid and nuclear data in *Camphora*, and we recommend enhanced taxonomic sampling and precise species identification to improve phylogenetic resolution and accuracy.

## 1. Introduction

The genera *Camphora* Fabr. and *Cinnamomum* Schaeff., both part of the Lauraceae sub-tribe Cinnamomineae (Laureae), are prominent members of one of the most ecologically significant woody plant families in moist tropical and subtropical forests globally [1]. Both genera comprise approximately two-hundred-and-fifty species, including forty-nine species in China, of which thirty are endemic and one has been introduced. Among these, *Cinnamomum aromaticum* Nees. is a well-known commercial source of cinnamon [2]. The genera *Camphora* has substantial economic value, serving as a primary source of camphor and essential oils (notably cinnamaldehyde, eugenol, and safrole), as well as providing spices from its bark, twigs, leaves, roots, flowers, and fruits, along with medicinal compounds, high-quality wood, and materials for perfumery and embalming [3]. Additionally, *Camphora* and *Cinnamomum* species play crucial ecological roles, given that most species in this clade are trees [4,5]. Both genera share distinctive features, including cymose inflorescences and often enlarged cupules that sometimes enclose the fruit partially. These traits complicate species delineation and identification, contributing to the group’s complex taxonomic history [6].

Several recent phylogenetic studies have highlighted the intricate relationships between *Camphora* and *Cinnamomum* taxa. In 2016, Huang observed that certain *Camphora* species, such as Ca. *chartophyllum* H. W. Li Y. Yang, Bing Liu & Zhi Yang. and Ca. *officinarum* Boerh. ex Fabr., were nested within different sections of *Cinnamomum* [7]. Similar findings were reported in 2017 by Rohwer, in 2020 by Song, and in 2022 by Xiao, who found that various *Camphora* species, including Ca. *tenuipile* (Kosterm) Y. Yang, B. Liu & Z. Yang, Ca. *chartophyllum*, and Ca. *officinarum*, appeared within sections of *Cinnamomum* [8,9,10]. These studies collectively indicated the polyphyletic taxa in *Camphora* and *Cinnamomum*, both morphologically and anatomically. A recent study in 2022 by Yang Zhi, based on an unusual phylogenetic tree of 76 chloroplast sequences, demonstrated the independent monophyly of *Camphora* and *Cinnamomum* as distinct groups, while also suggesting close relationships with *Sassafras* J. Presl and *Ocotea* Aubl. [1]. Clarifying the phylogenetic relationships within the subtribe Cinnamomineae and delineating these genera remains a substantial taxonomic challenge.

In order to better develop and utilize the existing resources of plants, the study of plant phylogeny and evolution can provide scientific theoretical reference and practical guidance. A chloroplast is an organelle within the cells that is autonomously inherited and possesses relatively independent and stable genetic material, so it is called the plastome. The plastome is a circular double-stranded genome that exhibits high sequence conservatism in terms of gene content and genome structural rearrangement. This conservatism is attributable to its special location and dependence on maternal inheritance. The plastome is characterized by a more stable structure and a moderate rate of molecular evolution [11,12]. Determination of genetically rich in information full-length plastome sequences and their comparative analysis has become one of the important and effective tools for plant phylogenetic studies [13,14].

Plant nuclear genomes are abundant in genetic information and play a crucial role in regulating plant morphology, as well as the majority of their physiological and biochemical functions within the organism. The nuclear genome is characterized by biparental inheritance, extensive variation in gene arrangement and copying, and other features, which render it uniquely suited for the identification of related species [15]. ITS (internal transcribed spacer) sequences are noncoding sequences located in the internal transcribed spacer region between 18S rDNA and 26S rDNA. Genetic studies of closely related species have demonstrated that ITS sequences typically contain substantial genetic information. This information can provide substantial support for the reconstruction of species phylogenies. Additionally, ITS sequences play a significant role in the formation of hybrids, polyploid origins, and other biological processes [16,17].

In this study, we re-evaluated the results of previous works [7,8,10]. We constructed a robust phylogenomic framework by integrating all available sequencing data from NCBI, and our database covered 90% of *Camphora* taxa. Using multiple topologies in phylogenetic comparative analyses, we account for variations in branch length and methodological differences in tree construction [18]. Based on these datasets, this study aims to address the following research questions:

1. What is the size range of *Camphora* plastomes?
2. What types of mutation events have occurred in the *Camphora* plastomes?
3. Are there highly variable regions in the plastomes of *Camphora*?
4. Is *Camphora* monophyletic based on chloroplast data?
5. If *Camphora* is monophyletic, how many distinct clades exist within the genus?

## 2. Results

### 2.1. Plastome Features Comparison

**Figure 1:**
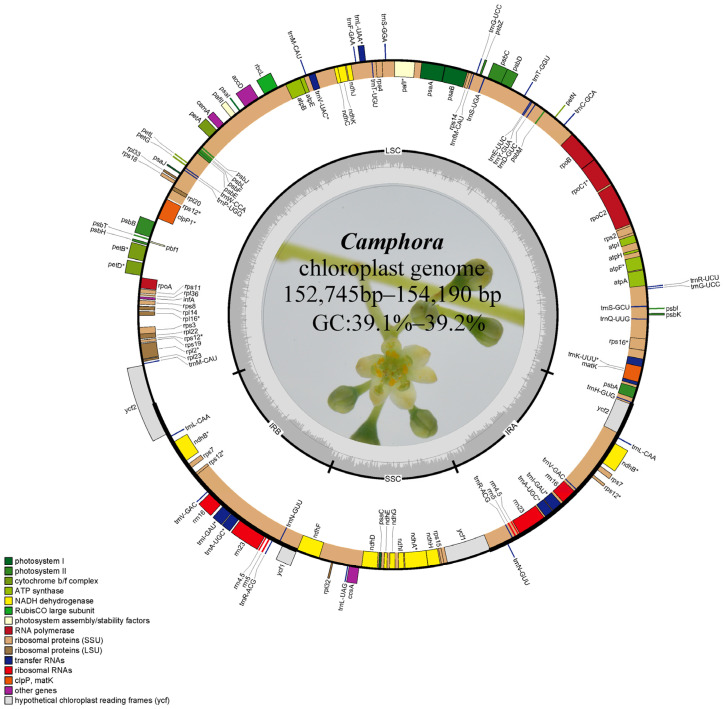
Circular gene map of *Camphora* taxa plastomes. Genes shown inside and outside the circle are transcribed in clockwise and counterclockwise directions, respectively. Genes belonging to different functional groups are color-coded. The GC and AT content are denoted by the dark gray and light gray colors in the inner circle, respectively. * indicates that the gene has introns.

Among the 22 species of *Camphora* selected for this study, the plastome length ranged from 152,745 bp in Ca. *longepaniculata* Y. Yang, B. Liu & Z. Yang. (with a GC content of 39.1%) to 154,190 bp in Ca. *longipetiolatum* H. W. Li Yu Song & Peiyao Xin (GC content of 39.2%) (Table 1). All plastomes displayed the typical angiosperm ring tetramer structure, comprising a large single-copy (LSC) region, a small single-copy (SSC) region, and two inverted repeat regions (IRa, IRb) (Figure 1). The LSC region’s length varied from 93,621 to 100,774 bp, while the SSC region’s length ranged from 18,855 to 18,968 bp. The IR region spanned from 16,901 to 20,810 bp. Across the genomes, 128 genes were identified, including 84 protein-coding genes, 8 rRNA genes, and 36 tRNA genes.

### 2.2. Characterization of Repeat Sequences and SSRs Analysis

**Figure 2:**
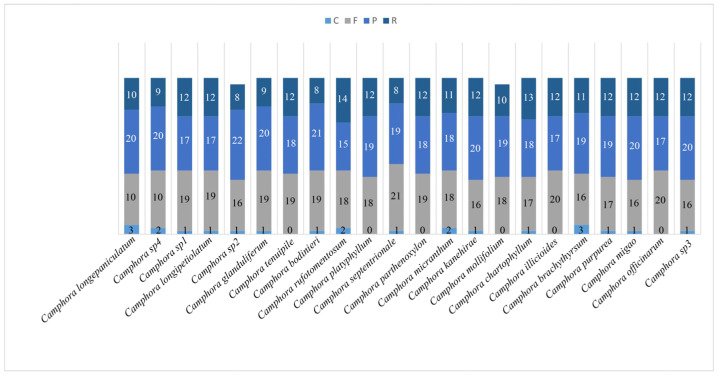
Repeats of the 22 newly sequenced plastomes. C represents complement repeats, F represents forward repeats, P represents palindromic repeats, and R represents reverse repeats.

Mutation serves as a fundamental driver of plant evolution, significantly affecting species identification. Repeat motifs are key contributors to mutational processes, including substitutions and indels. In the plastome of *Camphora*, 1074 repeats (including those in the IR region) were identified using the REPuter program (Figure 2). These repeats consist of 395 forward repeats (16–21 bp), 413 palindromic repeats (15–22 bp), 243 reverse repeats (8–14 bp), and 23 complement repeats (1–3 bp). An analysis of repeat elements in the 22 newly assembled plastomes revealed only minor variation in repeat element counts across different plastomes. Ca. *mollifolia* (H. W. Li) Y. Yang, B. Liu & Z. Yang exhibited the fewest repeat elements, totaling just 47 repeat units.

**Figure 3:**
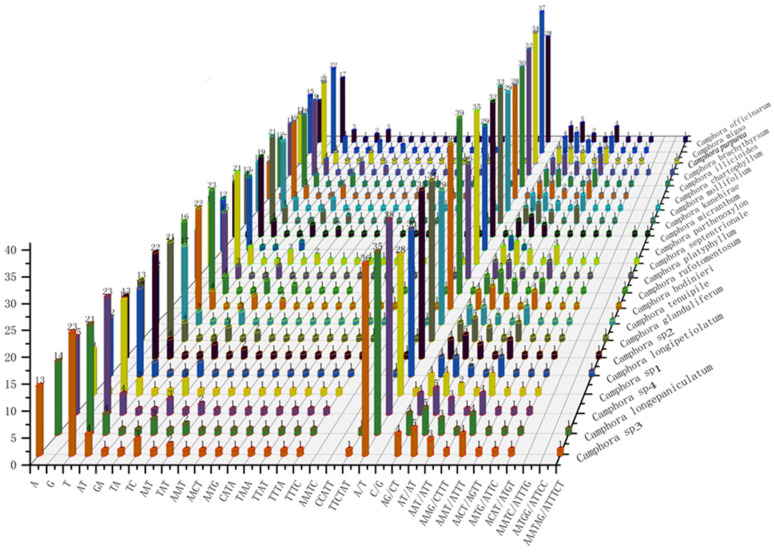
Statistics of SSR loci for plastomes of 22 species in the genus *Camphora.* The left vertical coordinate represents the number of SSR loci, the right vertical axis and different color columns represent different species in the genus *Camphora*, and the horizontal axis represents different single nucleotide repeat sequences.

Simple sequence repeats (SSRs) generally exhibit higher mutation rates than other neutral DNA regions due to slipped-DNA-strand formation. As non-recombinant, haploid, and uniparentally inherited markers, SSRs are frequently used in genetic, ecological, and evolutionary studies of plant populations (Figure 3). In the 22 newly assembled plastomes, 1130 SSRs were identified. Mono- and dinucleotide repeats were present across all *Camphora* plastomes, while a single pentanucleotide repeat was observed only in Ca. *micrantha* (Hayata) Y. Yang, B. Liu & Z. Yang and Ca. *brachythyrsa* (J. Li) Y. Yang, B. Liu & Z. Yang. A/T mononucleotide repeats were the most prevalent, aligning with previous findings that A/T mononucleotides are predominant in plastome SSRs.

### 2.3. Sliding-Window Analysis of the Plastomes

**Figure 4:**
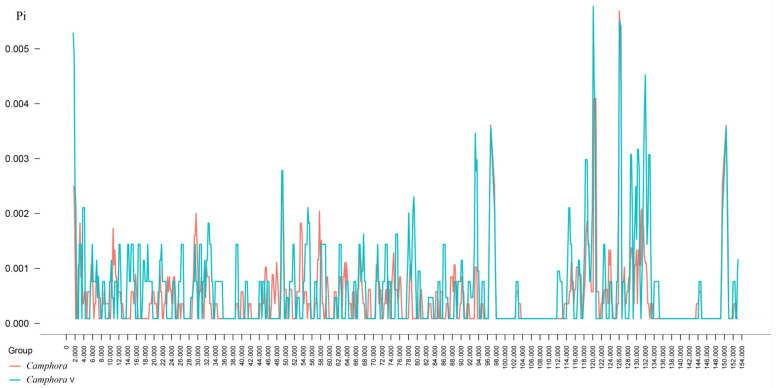
Nucleic acid variability in 22 species of the genus *Camphora*. The vertical axis indicates the nucleotide polymorphism value (Pi), and the horizontal axis indicates the sequence length. *Camphora* V represents the species of *Camphora* classified in the genus *Cinnamomum*.

SNP and indel markers tend to cluster within genomic divergence hotspots rather than being randomly distributed. In comparisons between *Camphora* and *Camphora V* taxa, these values ranged from 0 to 0.05959, with an average of 0.001826 (Figure 4). In *Camphora*, values ranged from 0 to 0.005706 (mean 0.00281), while in *Camphora V*, values spanned from 0 to 0.05959, with a mean of 0.00265. These findings indicate that intergeneric variation is greater than intrageneric variation. Additionally, nucleotide variation between the two genera is minimal, with plastome variation in the *Camphora* taxa primarily concentrated in the LSC and SSC regions, while sequences in the IR region exhibit low nucleotide variability.

### 2.4. Contraction and Expansion Analysis of IR Regions

**Figure 5:**
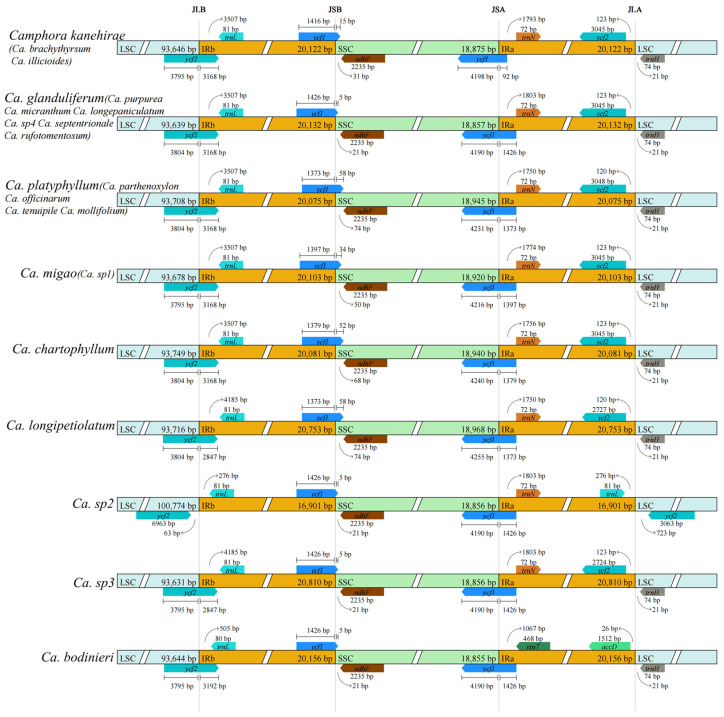
Visualization of the boundaries of the IR region of chloroplast genes in 22 species of the genus *Camphora*.

The LSC/IRb boundaries in the plastomes of the 22 *Camphora* taxa are relatively conserved (Figure 5), typically positioned within the coding region of the *ycf2* gene. The only exception is the genome of one suspected novel species *Camphora sp2*, where the LSC/IRb boundary lies in the noncoding region to the right of *ycf2*. The IRb/SSC and SSC/IRa boundaries are consistently located in the coding region of *ycf1*. Notably, *Camphora sp2* lacks the *trnH* gene, while in other *Camphora* taxa, the IRa/LSC boundary is situated in the noncoding region to the left of *trnH*.

### 2.5. Phylogenetic Analyses Based on Plastomes Sequences of Camphora Taxa

**Figure 6:**
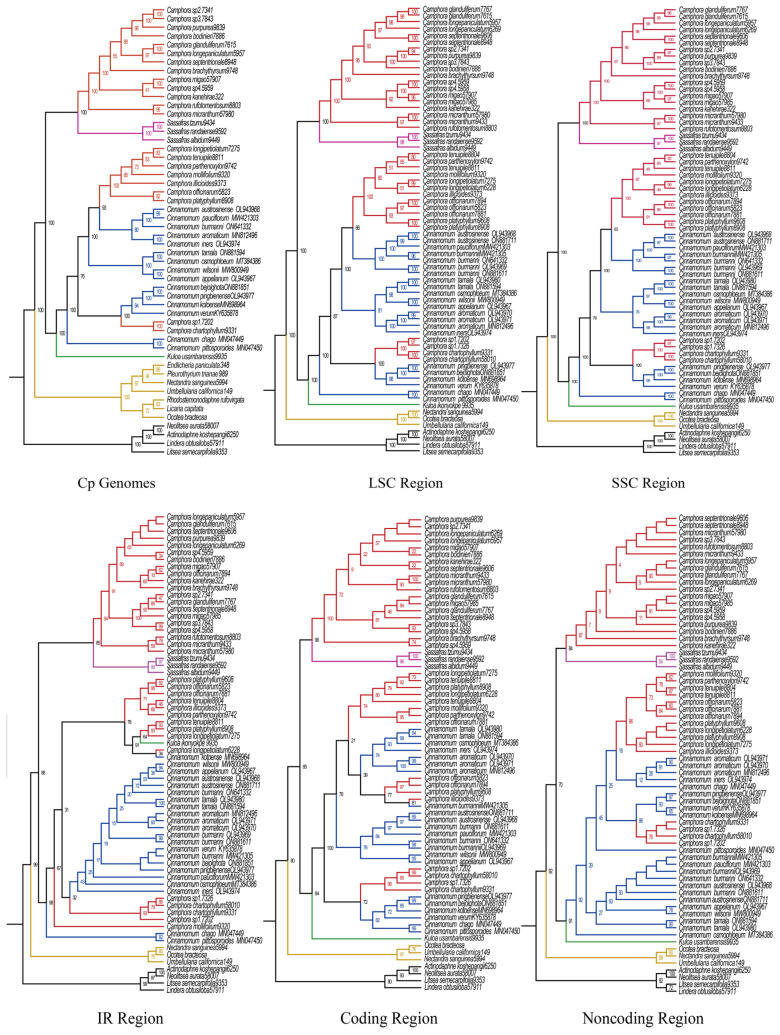
Phylogenetic tree based on plastome sequences. (**Cp**) Phylogenetic tree inferred from maximum likelihood analysis based on concatenated plastome sequences. (**LSC**) LSC region. (**SSC**) SSC region. (**IR**) IR region. (**Coding**) Coding region. (**Noncoding**) Noncoding region. *Lindera obtusiloba*, *Litsea semecarpifolia, Neolitsea aurata and Actinodaphne koshepangii* were used as the outgroup. The number on each node is a bootstrap support value. The evolutionary branch length represents the degree of branch variation. Different tribal clades are highlighted with different colors. The number after the species name is the accession number. One-thousand bootstrap replicates were conducted to obtain node support.

Two distinct data matrices were assembled and analyzed using maximum likelihood (ML), Bayesian inference (BI), and Astral methods. Matrix I included 52 complete plastomes, comprising 22 newly assembled plastomes and 30 additional taxa. The ML, BI, and Astral analyses of the complete plastomes sequences identified seven well-supported clades within the subtribe *Cinnamomeae* Kosterm., which includes four outgroup species. The first clade, nearest to the outgroup, comprises American species from seven genera *(Ocotea* Aubl., *Umbellularia* Nutt., *Rhodostemonodaphne* Rohwer & Kubitzki., *Endlicheria* Nees., *Licaria* Aubl., *Pleurothyrium* Nees., *Nectandra Rol. ex Rottb.*). The second clade includes *Camphora* and *Sassafras* J. Presl species (*S. albidum* (Nutt.) Nees., *S. randaiense* (Hayata) Rehder., *S. tzumu* (Hemsl.) Hemsl., Ca. *rufotomentosa* (K. M. Lan) Y. Yang, B. Liu & Z. Yang., Ca. *micrantha*, Ca. *septentrionalis* (Hand.-Mazz.) Y. Yang, B. Liu & Z. Yang., Ca. *glandulifera* (H. G. Ye & F. G. Wang) Y. Yang, B. Liu & Z. Yang., Ca. *longepaniculata*, Ca. *brachythyrsa*, Ca. *kanehirae* (Hayata) K. F. Chung & C. L. Hsieh, Ca. *bodinieri* (H. Lév.) Y. Yang, B. Liu & Z. Yang., Ca. *purpurea*, Ca. *sp3*, Ca. *sp2*, Ca. *migao*, Ca. *sp4*). The third clade represents the species *Kuloa ikonyokpe* F. Ritter. The fourth clade includes two *Cinnamomum* taxa *Ci. Chago* B. S. Sun & H. L. Zhao. and *Ci. Pittosporoides* Hand.-Mazz. The fifth clade includes both *Camphora* and *Cinnamomum* taxa Ca. *chartophylla*, Ca. *sp1*, *Ci. Verum* J. Presl., *Ci. Kotoense* Kaneh. & Sasaki., *Ci. Pingbienense* H. W. Li., and *Ci. Bejolghota* (Buch.-Ham.) Sweet. The sixth clade comprises six *Cinnamomum* species (*Ci. Iners* (Reinw. ex Nees & T. Nees) Blume, *Ci. Cassia* (L.) J. Presl, *Ci. Appelianum* Schewe., *Ci. Wilsonii* Gamble., *Ci. Osmophloeum* Kaneh., and *Ci. Tamala* (Buch.-Ham.) T. Nees & C. H. Eberm.). Lastly, the seventh clade also includes both *Camphora* and *Cinnamomum* taxa *Ci. Burmannii* (Nees & T. Nees) Blume., *Ci. Pauciflorum* Chun ex Hung T. Chang., *Ci. Austrosinense* H. T. Chang., Ca. *platyphylla*, Ca. *officinarum*, Ca. *illicioides*, Ca. *longipetiolatum*, Ca. *mollifolia*, Ca. *parthenoxylon*, and Ca. *tenuipilis* (Figure 6).

To evaluate potential conflicts among regions, we divided Matrix I into five subsets: large single copy (LSC) (Matrix VI), small single copy (SSC) (Matrix VII), inverted repeat (IR) (Matrix VIII), coding regions (Matrix IX), and noncoding regions (Matrix X). These matrices were analyzed using a maximum likelihood (ML) method. The topologies obtained from the IR regions, noncoding regions, and SSC regions were highly similar, with only subtle differences in the clades (Figure 6).

**Figure 7:**
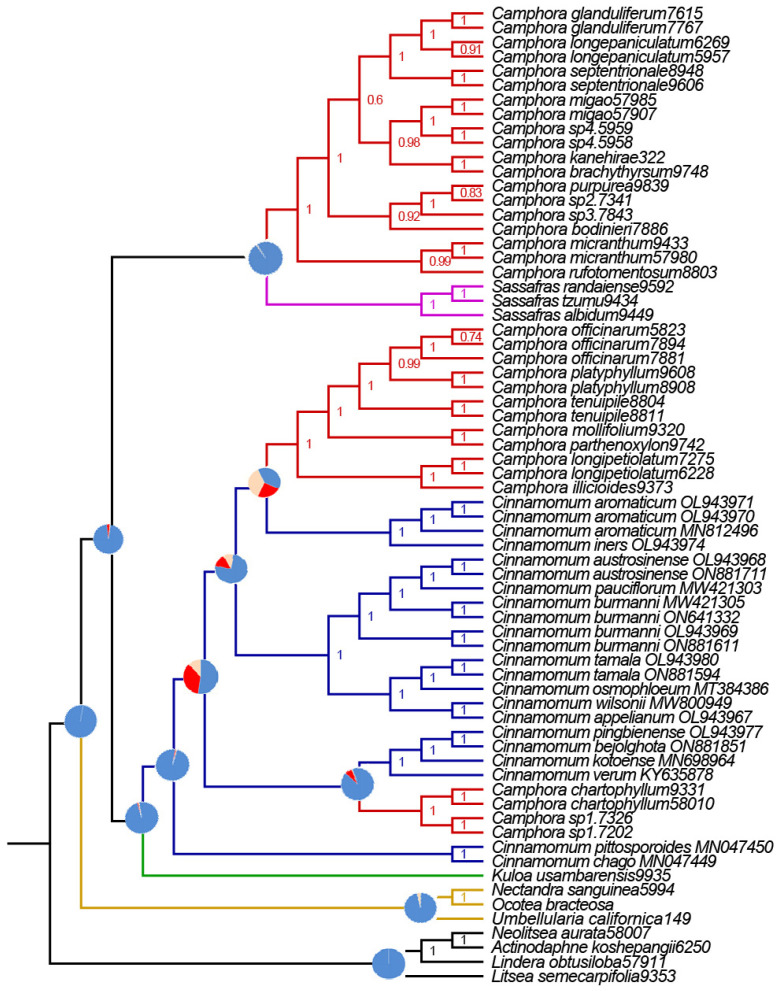
The plastome phylogenetic tree constructed via the Astral method. Pie charts for major clades representing the proportion of gene trees supporting the Astral species tree topology (blue), the main alternative bifurcation (red), and the remaining alternatives (yellow). The QS scores are shown in the branches.

The phylogenetic analysis of Matrix V (using Astral methods) was most successful in reconstructing the evolutionary relationships in the *Cinnamomum* and *Camphora* groups, as well as their relationships with closely related outgroups. Our phylogenies from Matrix V are consistent with those from Matrices I, II, VI, VII, and X. Seven well-supported monophyletic groups were recovered in the equally strongly supported *Cinnamomum* and *Camphora* clades (Figure 7).

### 2.6. Phylogenetic Analysis Based on Lauraceae nrDNA Genome Sequences

Data matrices III and IV were assembled and analyzed using both maximum likelihood (ML) and Bayesian inference (BI) methods. Matrix III included 52 complete nrDNA genomes, comprising members from *Litsea Lam.*, *Lindera Thunb.*, *Actinodaphne* Nees., *Neolitsea* Merr., *Ocotea*, *Umbellularia*, *Rhodostemonodaphne*, *Endlicheria*, *Licaria*, *Pleurothyrium*, *Kuloa* Trofimov & Rohwer, *Sassafras*, *Camphora*, *Nectandra Rol. ex Rottb.*, and *Cinnamomum*. *Litsea semecarpifolia* served as the outgroup. Matrix IV included 52 Lauraceae taxa, comprising 22 complete plastomes and 30 additional taxa, with *Lindera obtusiloba*, *Litsea semecarpifolia, Neolitsea aurata and Actinodaphne koshepangii* were again used as the outgroup.

**Figure 8:**
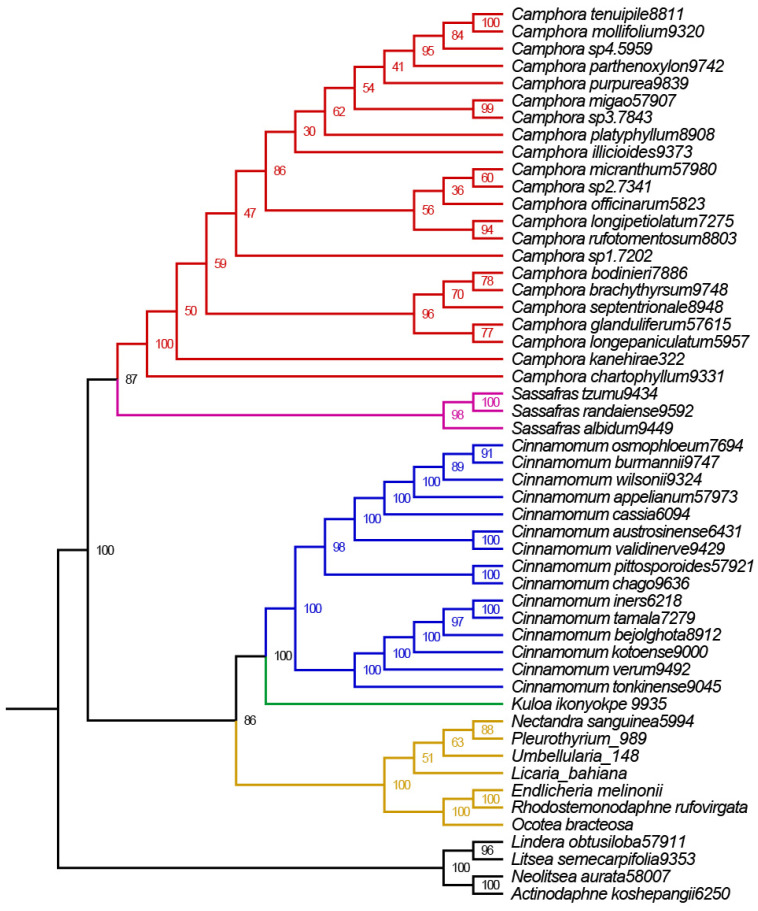
Phylogenetic tree based on nrDNA sequences. *Lindera obtusiloba*, *Litsea semecarpifolia, Neolitsea aurata and Actinodaphne koshepangii* were used as the outgroup. The number on each node is the bootstrap support value. The evolutionary branch length represents the degree of branch variation. Different tribal clades are highlighted with different colors. The number after the species name is the accession number. One-thousand bootstrap replicates were conducted to obtain node support.

Both BI and ML analyses revealed six highly supported clades in the subfamily Lauraceae. The first clade, closest to the root of the tree, consists of the tribe *Litsea*, which includes *L. semecarpifolia* (Wall. ex Nees) Hook. f., *L. obtusiloba*, *A. koshepangii* Chun ex Hung T. Chang., and *N. aurata*. The second clade encompasses the group *Ocotea bracteosa*, *Pleurothyrium*, *Nectandra sanguinea*, *Licaria bahiana*, *Rhodostemonodaphne rufovirgata*, *Endlicheria melinonii*, and *Umbellularia*, which are closely related and show high support values in the plastome phylogenetic tree. The third clade is represented by the group *Kuloa*, with only one species in our sample (*Kuloa ikonyokpe* F. Ritter). The fourth clade consists of the tribe *Cinnamomum*, while the fifth clade includes three species (*Sassafras albidum*, *Sassafras randaiense*, *Sassafras tzumu*), which do not group with the other tribes. The sixth clade represents the group *Camphora* (Figure 8).

## 3. Discussion

### 3.1. Changes in Genomic Characteristics and Gene Content

In this study, we sequenced the complete plastomes of 22 *Camphora* taxa using Illumina sequencing technology. The plastomes of these *Camphora* taxa are highly similar to those of other sequenced plastomes in the genus Lauraceae [1,7,9]. The analyzed plastomes ranged in size from 152,745 to 154,190 bp, with a size difference of 1445 bp. Each genome features a large single-copy (LSC) region and a small single-copy (SSC) region, separated by two short inverted repeat (IR) regions (Figure 1). Comparative genomic analysis identified two primary reasons for the observed size differences. First, one copy of the *ycf2* gene, which is typically 6963 bp long, was incomplete in all *Camphora* taxa LSC regions, reduced to 3795 bp. However, a complete copy was found in *Camphora sp3*, contributing approximately 3168 bp to the length difference. Second, the lengths of the *ycf1* genes copied to the IRa region varied from 1426 bp to 92 bp among the 22 plastomes, accounting for approximately 1334 bp of the size variation. The *ycf1* and *ycf2* genes are located at the boundaries between the IR regions and the LSC or SSC regions (Figure 3), with these length mutations contributing to the contraction of the IR regions in the plastome [19]. Notably, *ycf2* exhibited higher genetic divergence among the 22 sequenced plastomes of the *Camphora* taxa compared to *ycf1* (Figure 4).

We also identified five highly variable loci, including *psbJ-psbL*, *ycf2*, and *ycf1*, which have previously been recognized as hypervariable regions in *Persea* Mill., *Phoebe* Nees., *Lindera* Thunb, and *Alseodaphne* Nees. [1,19,20,21]. Additionally, the fragments *trnK (UUU)* and *ccsA-ndhD* appeared to be especially variable loci in the *Camphora* plastomes, demonstrating promising levels of variation for applications in DNA barcoding or intraspecific studies. Although overall genetic divergence was low, nearly ten divergence hotspots and repeat mutations were identified.

### 3.2. Characterization of Repeat Sequences and SSRs of Camphora

During the lengthy process of genetic evolution in plants, long repetitive sequences have contributed to the structural rearrangement of the plastome, which in turn has contributed to the increase in the genetic diversity of the species population [22]. In the plastome of *Camphora*, 1074 repeats (including those in the IR region) were identified. The majority of these repeats are forward and palindromic, a finding that aligns with the majority of plant studies, thereby suggesting that *Camphora* possesses a substantial genetic foundation [23]. SSR is frequently regarded as the optimal choice for studies in population genetics, species identification, and fingerprinting because of its highly conserved, uniparental inheritance, rich in polymorphism, stable, and reliable sequencing results [24]. Based on the SSR analysis of 22 *Camphora* plastomes, a total of 1130 SSR loci were identified. Mono- and dinucleotide repeats were present across all *Camphora* plastomes, while a single penta-nucleotide repeat was observed only in Ca. *micrantha* and Ca. *brachythyrsa*. In addition, as in most angiosperms reported, A/T single-nucleotide repeats are the most prevalent in *Camphora*, indicating that the sequence of the plastomes of *Camphora* prefers A/T bases [25]. Based on the SSR analysis of the plastomes of *Camphora*, the plastomes of *Camphora* are rich in SSR polymorphism information, which can provide a reference for the identification of germplasm resources and evolutionary analysis of *Camphora*.

### 3.3. Phylogenetic Relationships Inferred from the Subtribe Cinnamomineae Plastome

**Figure 9:**
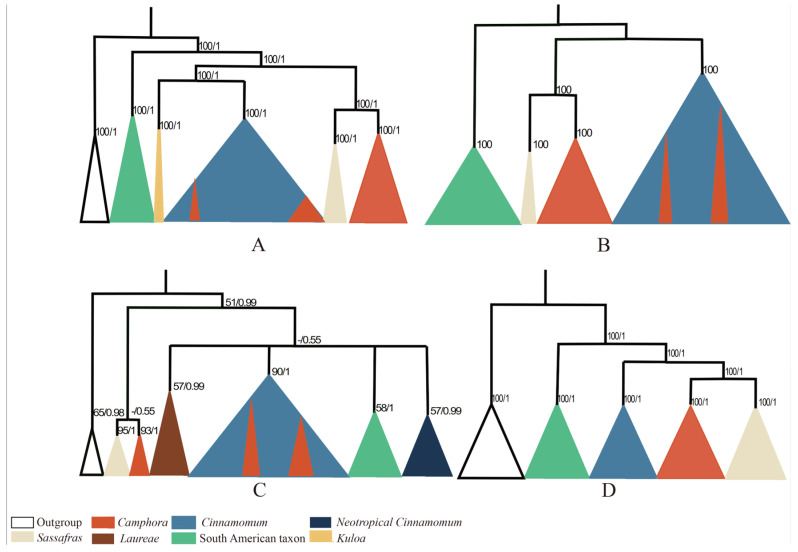
Phylogenetic tree based on plastome sequences. (**A**) Phylogenetic tree inferred by based on maximum likelihood analysis of plastome sequences. (**B**) Phylogenetic tree inferred by Xiao et al. based on maximum likelihood analysis of plastome sequences. (**C**) Phylogenetic tree inferred by Rhode et al. based on maximum likelihood analysis of plastome sequences. (**D**) Phylogenetic tree inferred by Yang et al. based on maximum likelihood analysis of plastome sequences.

Our plastome phylogenomic results confirm that the subtribe *Cinnamomineae* contains seven major clades. The methods of data analysis (maximum likelihood [ML], Bayesian inference [BI], and Astral) did not significantly affect the phylogenetic trees, as the resulting topologies were highly similar across each dataset. The phylogenies based on the large single-copy (LSC), small single-copy (SSC), inverted repeat (IR), coding regions, and noncoding regions exhibited overall congruent topologies, except for the *Kuloa* clade, which is one of the most complex. This suggests that there is no conflict among the plastome partitions. Huang et al. presented a Bayesian consensus tree based on ITS, *LEAFY*, and *RPB2* data, which recovered three well-supported monophyletic groups within the equally strongly supported *Cinnamomum* group [7]. Clade 1 included nearly all the sampled Asian *sect*. *Camphora* samples, while the American *Cinnamomum* species, along with *M. cinnamomoidea* and three *Aiouea* samples, formed Clade 3. All remaining ingroup Asian samples, six Australian samples, and African *O. ikonyokpe* formed Clade 2. Xiao et al. inferred a phylogenetic tree using maximum likelihood analysis based on concatenated protein-coding genes (PCG-c), which identified three major clades in the subtribe *Cinnamomineae* [9]. In Clade 1, *Nectandra* and *Ocotea* were sister to *Sassafras* and *Cinnamomum* (Clade 2). In Clade 2, nine of the twelve species from *Cinnamomum sect*. *Camphora* formed a monophyletic group that was sister to *Sassafras*. Clade 3 included the other three species (*C. chartophyllum*, *C. camphora*, and *C. tenuipile*) from *sect. Camphora*, nested in eighteen species from *sect. Cinnamomum*. The phylogenetic trees based on different data matrices produced similar relationships, and our topologies were consistent with those of Huang et al. and Xiao et al. Yang et al. conducted a new phylogenetic analysis of the *Cinnamomum* group using all available complete plastomes. Their plastome phylogenetic tree revealed four ingroup clades: Clade 1 included the American genera of the *Cinnamomum–Ocotea* complex; Clade 2 comprised *sect. Camphora s.s*.; Clade 3 contained the deciduous genus *Sassafras*; and Clade 4 included *sect. Cinnamomum* s.l. Our topology differed from that of Yang et al. [8] (Figure 9).

### 3.4. Phylogenetic Relationships Inferred from the Subtribe Cinnamomineae nrDNA

All maximum likelihood (ML) and Bayesian inference (BI) analyses of the nrDNA sequences fully resolved the phylogenetic relationships between *Camphora* and *Cinnamomum*, confirming their respective monophyly as reported by previous studies [1,7,8]. Five phylogenetically meaningful clades were identified among the deep lineages in our study. First, three *Sassafras* taxa and twenty-two *Camphora* taxa clustered into a single unit, with the twenty-two *Camphora* taxa representing the most comprehensive range available in the *Flora of China*. This finding aligns with Yang’s nrITS-based results [8], where three species of *Sassafras* and twelve *Camphora* taxa also formed a single clade, but it contradicts Rohde’s ITS-based results [9], which indicated that two *Sassafras* taxa and seven *Camphora* taxa each formed monophyletic lineages, with *Sassafras* diverging prior to *Camphora*.

Second, a single species of *Kuloa* from Africa clustered with fifteen *Cinnamomum* taxa, which encompass sequences of all available *Cinnamomum* taxa in NCBI. This is consistent with Yang’s finding that two *Kuloa* taxa and sixty-four *Cinnamomum* taxa formed a single clade [8]. Finally, the most complex and variable clade consists of *Ocotea* species from America, which emerged as a sister taxon to *Kuloa* and *Cinnamomum* in our analysis. This result contrasts with Huang’s findings [7], which positioned *Ocotea ikonyokpe* as the sister taxon to *Cinnamomum* based on ITS, LEAFY, and RPB2 data. Additionally, Rohde found that *Ocotea* and *Cinnamomum* formed separate monophyletic groups [9], with *Cinnamomum* diverging before *Ocotea*. This differs from Yang’s result [8], which suggested that Ocotea is the sister taxon of *Sassafras* and *Camphora*.

The topology obtained indicates that nrDNA sequences, when appropriately sampled, can provide robust and well-supported relationships among the deep lineages of *Camphora* and *Cinnamomum*. Although the topologies derived from different studies were not in complete agreement, both *Camphora* and *Cinnamomum* were consistently divided into monophyletic taxa. We speculate that the primary reason for the inconsistencies in topology is related to the abundance and evenness of the sampled species [26,27,28].

### 3.5. Phylogenetic Incongruence in the nrDNA and Plastome

**Figure 10:**
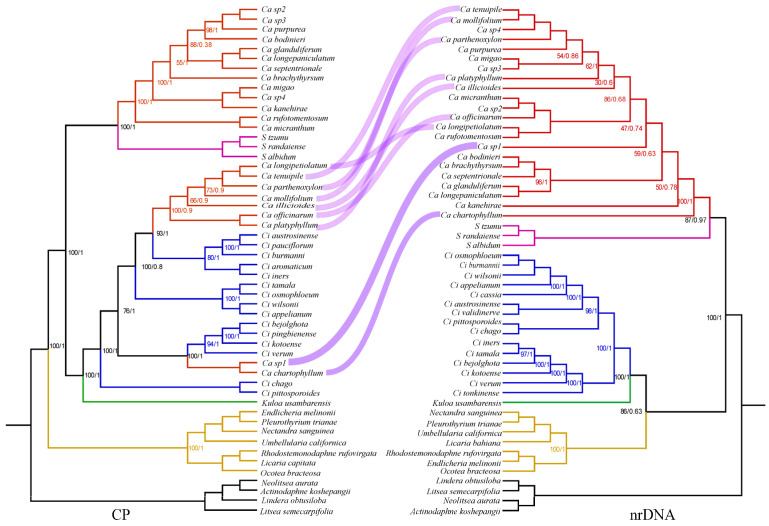
The phylogenetic trees based on the maximum likelihood method show plastome sequences result on the left and nrDNA result on the right. The purple lines indicate the emergence of conflicting topologies in phylogenetic analyses of nrDNA sequences and plastome sequences. The number on each node is bootstrap support value. The evolutionary branch length represents the degree of branch variation. Different tribal clades are highlighted with different colors. The number after the species name is the accession number. One-thousand bootstrap replicates were conducted to obtain node support.

Phylogenetic analyses of both nrDNA and plastome datasets reveal a clear signal that contradicts traditional generic classifications. The species currently classified under *Camphora* do not form a monophyletic group; instead, they are distributed across three distinct clades. Two of these clades are nested in the genera *Cinnamomum* (clades 5 and 7), as evidenced in the plastome dataset (Figure 10). Notably, conflicting topologies arise from the analyses of the separate nrDNA and plastome datasets. The third clade (clade 2) comprises most species of *Camphora* examined in this study, as well as species from the genera *Sassafras*.

**Figure 11:**
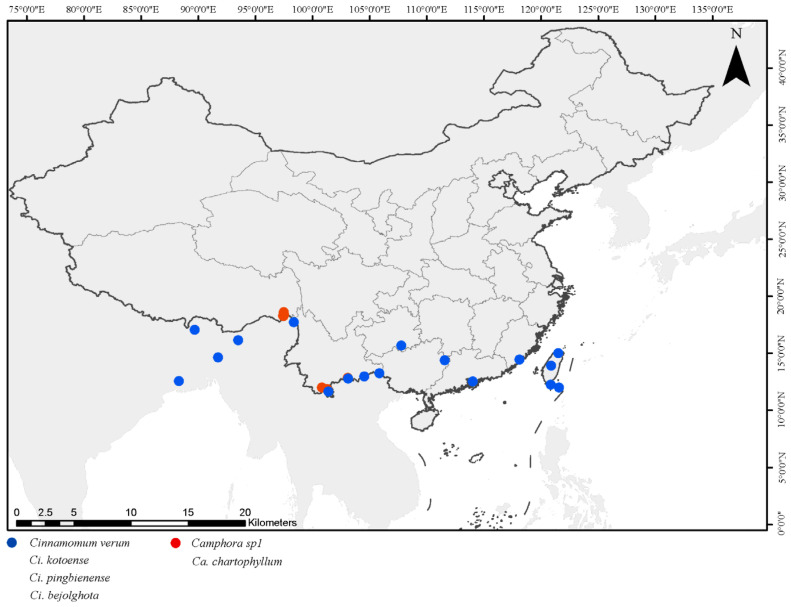
Distribution of the first nucleoplasmic conflict group.

**Figure 12:**
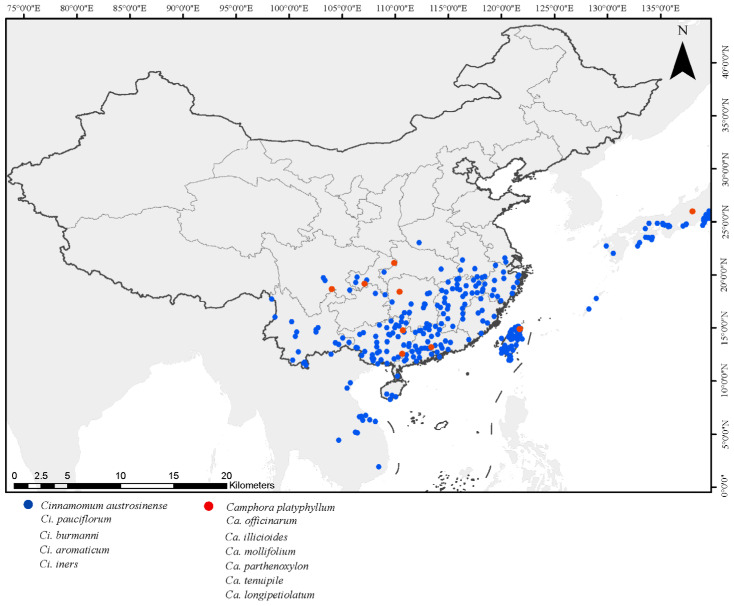
Distribution of the second nucleoplasmic conflict group.

The observed nuclear–cytoplasmic discrepancy is commonly encountered in studies of interspecific relationships [29,30,31]. Such discrepancies may arise from biological convergence, rapid diversification, pedigree recombination, and heterozygous osmosis, with heterozygosity being a significant factor [30,31,32]. Thus, the nuclear-cytoplasmic inconsistency between *Camphora* and *Cinnamomum* taxa in this study could be attributed to hybridization. Specifically, the flowering periods of the genera *Cinnamomum* coincide with or follow those of *Camphora*, allowing for potential pollen transfer from *Camphora* to *Cinnamomum* [33,34] (Figure 11 and Figure 12). Additionally, our field investigations indicate overlapping distribution areas between these genera, further suggesting the possibility of hybridization [35,36] (Table 2).

Hybridization typically occurs during the reproductive process, necessitating overlapping flowering periods and breaking geographical isolation. Chloroplast capture represents an extreme form of hybridization, wherein a new species is formed through interspecific hybridization and subsequent backcrossing, resulting in a species with the cytoplasm of one species and the nucleus of another [37]. Given the genetic characteristics of plastome genes—complete non-recombination and minimal selection pressure on conserved housekeeping sites [38,39]—gene infiltration into plastome is generally more prevalent than into nuclei.

Chloroplast capture events have been documented across various plant taxonomic groups [40,41,42], often accompanied by a significant lack of nuclear gene penetration during parental inheritance [40,43], even in extensive genomic surveys [44]. Based on previous research, we speculate that chloroplast capture events between *Cinnamomum* and *Camphora* may have resulted from ancient hybridization events [3,45,46,47]. We propose that at least two chloroplast capture events have occurred between these genera during their evolutionary history. The first event likely took place before the differentiation of *Cinnamomum*, with Ca. *chartophyllum* and Ca. *sp1* capturing plastomes from *Cinnamomum verum*, among others. The second event occurred after the divergence of the genera *Camphora*, involving species such as Ca. *longipetiolatum*, Ca. *tenuipilis*, Ca. *parthenoxylon*, Ca. *mollifolia*, Ca. *illicioides*, Ca. *officinarum*, and Ca. *platyphylla*, which captured chloroplast genomes from *Ci. burmanni*, among others.

### 3.6. Insights to the Phylogenetic Reconstruction Using Genomes Camphora Plastome

Among the four widely used plastome markers in plant phylogenetic inference (i.e., *matK*, *rbcL*, *ycf1*, *ycf2*), we identified one seldom-used locus, *ccsA-ndhD*, that outperformed the commonly used markers, suggesting its potential as a new candidate marker. The completeness of species sampling can significantly impact phylogenetic structure. For instance, Xiao and Ge (2022) included 10 species in their phylogenetic analysis of *Camphora* and *Cinnamomum* [10], while Yang et al. (2023) included 12 species [8]. Additionally, the absence of certain non-human historical events in species sampling can compromise the robustness of the phylogenetic tree. Zhang et al. (2023) erroneously concluded that Sassafras was transmitted from South America to Australia due to a lack of North American species in their analysis [48].

Furthermore, issues such as missing data and false positives in genomic sequences can adversely affect the topology of phylogenetic trees by resulting in a significant loss of identifiable sites. Conflicts in phylogenetic structures often arise from stochastic inferences based on genes with low informational content. The choice of assembly strategy also influences the resulting phylogenetic tree; genome assembly typically employs two algorithms: Overlap–Layout–Consensus (OLC) and De Bruijn Graph (DBG). Variations in parameter choices can lead to different final sequence datasets, as demonstrated by Xiao and Ge (2022), who assembled an additional segment of data for Ca. *chartophylla* [10]. Finally, the selection of outgroups is crucial for constructing a robust phylogenetic tree, necessitating careful consideration to ensure accurate phylogenetic topology.

## 4. Materials and Methods

### 4.1. Taxon Sampling

A total of 35 accessions representing 22 species of the genus *Camphora* were included (Table 3). Additionally, 22 plastomes sequences of 15 species of the genus *Cinnamomum* and 13 plastomes sequences of related genera were also downloaded from NCBI, encompassing nearly the entire distribution range of the group [47]. Four core species of the Lauraceae tribe were selected as outgroups based on prior studies [49].

### 4.2. Plastome Assembling and Annotation

Sequencing was conducted on an Illumina HiSeq 2000 at BGI-Shenzhen in Guangdong, yielding over 4 Gb of sequence data for each sample. Paired-end reads were filtered using GetOrganelle 1.7.5.0 software [50], with parameters set as follows: word size -w 120, rounds -R 10, k-mer -K 75 to 105, and pregrouping-P 300,000. Genome maps were generated with OGDRAW v1.3.1 [51]. Multiple alignments were performed using MAFFT v7.520 [52] and manually adjusted as necessary. The plastome sequences were annotated with Geneious v8.1.3 software [53]. Correlations among these parameters were analyzed using Pearson’s correlation coefficient reporting and r^2^-values. The annotated plastome sequences were submitted to the Lauraceae Chloroplast Genome Database (https://lcgdb.wordpress.com/home/species/, accessed on 21 January 2025).

### 4.3. Repeat Elements and Simple Sequence Repeats

Both direct and inverted repeats were analyzed using REPuter (https://bibiserv.cebitec.uni-bielefeld.de/reputer, accessed on 10 January 2024), which identifies direct, reverse, and palindromic repeats in the plastome. For repeat identification, the following parameters were set in REPuter: (i) a minimum repeat size of 30 bp, and (ii) a sequence identity of 90% or greater, based on a Hamming distance of 3 [54]. Simple sequence repeats (SSRs) were predicted using MISA (https://webblast.ipk-gatersleben.de/misa/index.php?action=1, accessed on 10 January 2024) [55,56], with the parameters established as follows: ten repeat units ≥ 10 for mononucleotide SSRs; five repeat units ≥ 5 for dinucleotide SSRs; four repeat units ≥ 4 for trinucleotide SSRs; and three repeat units ≥ 3 for tetranucleotide, pentanucleotide, and hexanucleotide SSRs.

### 4.4. Sliding Window Analysis of the Plastomes

MAFFT v7.520 [53] was used to align the 72 plastome sequences of *Camphora* and *Cinnamomum*. After manual adjustments with BioEdit v7.0.9.0 [57], we conducted a sliding window analysis to calculate nucleotide variability and pairwise nucleotide divergence across the plastomes using DnaSP v6 [58]. We defined the window length as 600 base pairs, and the step size as 200 base pairs, with the resulting values plotted in R. Additionally, we used CPJSdraw v1.158 [59] to analyze the expansion and contraction of the SC/IR boundaries in the chloroplast structure across the four major compartments of 22 *Camphora* taxa.

### 4.5. Phylogenetic Analyses

Data matrices (I, II, III, IV) were assembled and analyzed using maximum likelihood (ML), Bayesian inference (BI), and Astral methods [60]. The matrices included 72 Lauraceae species, comprising 35 species from *Camphora* and *Cinnamomum* as well as 37 species from other genera. To evaluate potential conflicts among regions, Matrix I was divided into five subsets: large single copy (LSC) (Matrix V), small single copy (SSC) (Matrix VI), inverted repeat (IR) (Matrix VII), coding regions (Matrix VIII), and noncoding regions (Matrix IX). These subsets were analyzed using the maximum likelihood (ML) method. Alignments were conducted in Mauve v2.4.0 and adjusted manually in Geneious v9.1.7 [53,61]. Bayesian inference was performed using MrBayes v3.2.6 [62], with the best-fit DNA substitution models selected via jModelTest v2.1.10 [63]. The optimal models for Markov chain Monte Carlo analysis were run in MrBayes v3.2.6 for one million generations, starting with a random tree and sampling every 1000 generations. The first 25% of trees were discarded as burn-in, and the remaining trees were used to generate a majority-rule consensus tree. Maximum likelihood analysis was executed using RAxML 7.2.6 [64], with the best-fit DNA substitution models for matrices I, II, III, and IV chosen for phylogenetic tree construction. One-thousand bootstrap replicates were conducted to obtain node support.

## 5. Conclusions

In this study, we newly sequenced 35 plastomes of 22 *Camphora* taxa and combined these with 22 published plastome sequences of 15 *Cinnamomum* taxa and other 15 taxa of closely related genera from the tribe Laureae to reconstruct the phylogenetic relationships of Cinnamomineae (Laureae). The plastomes of the genera *Camphora* demonstrated high levels of conservation and similarity, with the presence of multiple mutation sites and SSR sites identified. This provides a robust theoretical foundation for further genetic variation analysis of *Camphora* taxa. Phylogenetic analyses showed that the plastome generally exhibited a consistent topology, and the nrDNA phylogenetic tree clearly distinguished *Camphora* and *Cinnamomum* taxa, suggesting that nrDNA sequences could provide a well-supported relationship between the two at a deeper level of genealogy. Hybridization and chloroplast capture are suspected to be primary causes of the discordanc observed between the plastome, nrDNA, and even mitochondrial genomes. Furthermore, employing diverse phylogenetic inference methods (such as concatenated and multi-species coalescent approaches) and ensuring extensive and homogeneous species sampling are key considerations for future phylogenomic analyses.

## Figures and Tables

**Table 1:** Sequence information of the 22 *Camphora* plastomes.

Taxa	Sequence Length (bp)	LSC Length (bp)	SSC Length (bp)	IR Legth (bp)	Gene Number	Number of Protein-Coding Genes	rRNA Gene Number	tRNA Gene Number	GC Content (%)
*Camphora bodinieri*	152,811	93,644	18,855	20,156	128	84	8	36	39.1
Ca. *officinarum*	152,819	93,706	18,963	20,075	128	84	8	36	39.1
Ca. *chartophylla*	152,851	93,749	18,940	20,081	128	84	8	36	39.1
Ca. *glandulifera*	152,760	93,639	18,857	20,132	128	84	8	36	39.1
Ca. *illicioides*	152,821	93,707	18,880	20,117	128	84	8	36	39.2
Ca. *longepaniculata*	152,745	93,621	18,860	20,132	128	84	8	36	39.1
Ca. *longipetiolatum*	154,190	93,716	18,968	20,753	128	84	8	36	39.2
Ca. *micrantha*	152,804	93,685	18,893	20,113	128	84	8	36	39.1
Ca. *migao*	152,804	93,678	18,920	20,103	128	84	8	36	39.1
Ca. *mollifolia*	152,813	93,701	18,962	20,075	128	84	8	36	39.1
Ca. *platyphyllum*	152,803	93,708	18,945	20,075	128	84	8	36	39.1
Ca. *parthenoxylon*	152,813	93,702	18,961	20,075	128	84	8	36	39.1
Ca. *septentrionalis*	152,814	93,652	18,898	20,132	128	84	8	36	39.1
Ca. *tenuipilis*	152,815	93,704	18,961	20,075	128	84	8	36	39.1
Ca. *kanehirae*	152,765	93,646	18,875	20,122	128	84	8	36	39.1
Ca. *rufotomentosa*	152,834	93,709	18,861	20,132	128	84	8	36	39.1
Ca. *brachythyrsa*	152,777	93,646	18,887	20,122	128	84	8	36	39.1
Ca. *purpurea*	152,746	93,626	18,856	20,132	128	84	8	36	39.1
Ca. *sp1*	152,845	93,743	18,902	20,100	128	84	8	36	39.1
Ca. *sp2*	153,432	100,774	18,856	16,901	128	84	8	36	39.1
Ca. *sp3*	154,107	93,631	18,856	20,810	128	84	8	36	39.1
Ca. *sp4*	152,826	93,700	18,862	20,132	128	84	8	36	39.1

**Table 2:** Overlapping plant flowering time statistics. E represents the first half of the month, and L represents the second half of the month. Jan: January, Feb: February, Mar: March, Apr: April, May: May, Jun: June, Jul: July, Aug: August, Sept: September. Pink represents the flowering period of *Camphor* species, while blue represents the flowering period of *Cinnamomum* species.

	E. Jan	L. Jan	E. Feb	L. Feb	E. Mar	L. Mar	E. Apr	L. Apr	E. May	L. May	E. Jun	L. Jun	E. Jul	L. Jul	E. Aug	L. Aug	E. Sept	L. Sept
Ca. *chartophylla*																		
Ca. *sp1*																		
*Ci. pingbienense*																		
*Ci. bejolghota*																		
*Ci. verum*																		
*Ci. kotoense*																		
*Ca.aillicioides*																		
Ca. *platyphylla*																		
Ca. *officinarum*																		
Ca. *parthenoxylon*																		
Ca. *tenuipilis*																		
*Ci. longipetiolatum*																		
Ca. *mollifolia*																		
*Ci. pauciflorum*																		
*Ci. burmannii*																		
*Ci. iners*																		
*Ci. cassia*																		
*Ci. austrosinense*																		

**Table 3:** Species collection of *Camphora.* Locality is the location where the species sample was collected, Voucher is the sample number, Accessron is the sequencing number, _ indicates that the species was collected in the article, and No indicates that the species was not collected in the article. All specimens were deposited in the Herbarium of Guangxi Normal University.

Taxa	Locality	Voucher	Accession	Xiao et al., 2022 [10]	Yang et al., 2022 [8]
*Camphora bodinieri*	Kunming, Yunnan	31227	7886	_	_
H. Lév. Y. Yang, Bing Liu & Zhi Yang					
*Camphora officinarum* Nees	Hangzhou, Zhejiang	35367	7881	_	_
	Changsha, Hunan	SY26	7894		
	Changsha, Hunan	37831	5823		
*Camphora foveolata*				_	_
Merr. Y. Yang, Bing Liu & Zhi Yang					
*Camphora chartophylla*	Menglun, Yunnan	YST12	58010	_	_
H. W. Li Y. Yang, Bing Liu & Zhi Yang		35955	9331		
*Camphora glandulifera*	Chayu, Xizang	X289	57615	_	_
Wall. Nees		35036	7767		
*Camphora illicioides*	Ledong, Hainan	34222	9373	no	no
A. Chev. Y. Yang, Bing Liu & Zhi Yang					
*Camphora longepaniculata*	Baoshan, Yunnan	34456	6269	_	no
Gamble Y. Yang, Bing Liu & Zhi Yang		35832	5957		
*Camphora longipetiolatum*	Mojiang, Yunnan	34492	6228	no	_
H. W. Li, Yu Song & Peiyao Xin	Kachin, Myanmar	34901	7275		
*Camphora micrantha*	Changsha, Hunan	37720	57980	_	_
Hayata Y. Yang, Bing Liu & Zhi Yang		37726	9433		
*Camphora migao*	Qiaoluan, Guizhou	36725	57985	_	no
H. W. Li Y. Yang, Bing Liu & Zhi Yang		XWB10	57907		
*Camphora mollifolia*	Daluo, Yunnan	SY1785	9320	no	_
H. W. Li Y. Yang, Bing Liu & Zhi Yang					
*Camphora philippinensis*				no	no
Merr. Y. Yang, Bing Liu & Zhi Yang					
*Camphora platyphyllum*	Baoshan, Yunnan	35035	9608	no	_
Diels Y. Yang, Bing Liu & Zhi Yang		32700	8908		
*Camphora parthenoxylon*	Manxing, Yunnan	34893	9742	no	_
Jack Nees					
*Cinnamomum saxatile*	Guilin, Guangxi	37787	6227	no	no
H. W. Li		35167	6127		
*Camphora septentrionalis*	Kunming, Yunnan	L0004	9606	_	_
Hand.-Mazz. Y. Yang, Bing Liu & Zhi Yang		SW9106	8948		
*Camphora tenuipilis*	Mengla, Yunnan	SY1672	8811	_	_
Kosterm. Y. Yang, Bing Liu & Zhi Yang		SY1778	8804		
*Cinnamomum kanehirae*	Taiwan, China	34424	N322	no	no
Hayata					
*Camphora rufotomentosa*	Daluo, Yunnan	34329	8803	no	no
K. M. Lan Y. Yang, Bing Liu & Zhi Yang					
*Camphora brachythyrsa*	Wuhan, Hubei	36142	9748	no	no
J. Li Y. Yang, Bing Liu & Zhi Yang					
*Camphora purpurea*	Wuhan, Hubei	XWB53	9839	no	no
H.G.Ye & F.G.Wang Y.Yang, Bing Liu & Zhi Yang					
*Camphora sp1*	Kachin, Myanmar	34492	7326	no	no
		34573	7202		
*Camphora sp2*	Kachin, Myanmar	34687	7341	no	no
*Camphora sp3*	Ailaoshang, Yunnan	35454	7843	no	no
*Camphora sp4*	Baoshan, Yunnan	35833	5958	no	no
		35834	5959		

## Data Availability

Data are contained within the article.
